# The OxyR-regulated *phnW* gene encoding 2-aminoethylphosphonate:pyruvate aminotransferase helps protect *Pseudomonas aeruginosa* from *tert*-butyl hydroperoxide

**DOI:** 10.1371/journal.pone.0189066

**Published:** 2017-12-07

**Authors:** Warunya Panmanee, Nisanart Charoenlap, Sopapan Atichartpongkul, Aekkapol Mahavihakanont, Matthew D. Whiteside, Geoff Winsor, Fiona S. L. Brinkman, Skorn Mongkolsuk, Daniel J. Hassett

**Affiliations:** 1 Department of Molecular Genetics, Biochemistry and Microbiology, University of Cincinnati College of Medicine, Cincinnati, OH; 2 Laboratory of Biotechnology, Chulabhorn Research Institute, Lak Si, Bangkok, Thailand; 3 Department of Molecular Biology and Biochemistry, Simon Fraser University, Burnaby, British Columbia, Canada; 4 Department of Biotechnology, Faculty of Science, Mahidol University, Bangkok, Thailand; University of Massachusetts Medical School, UNITED STATES

## Abstract

The LysR member of bacterial transactivators, OxyR, governs transcription of genes involved in the response to H_2_O_2_ and organic (alkyl) hydroperoxides (AHP) in the Gram-negative pathogen, *Pseudomonas aeruginosa*. We have previously shown that organisms lacking OxyR are rapidly killed by <2 or 500 mM H_2_O_2_ in planktonic and biofilm bacteria, respectively. In this study, we first employed a bioinformatic approach to elucidate the potential regulatory breadth of OxyR by scanning the entire *P*. *aeruginosa* PAO1 genome for canonical OxyR promoter recognition sequences (ATAG-N_7_-CTAT-N_7_-ATAG-N_7_-CTAT). Of >100 potential OxyR-controlled genes, 40 were strategically selected that were ***not*** predicted to be involved in the direct response to oxidative stress (e.g., catalase, peroxidase, etc.) and screened such genes by RT-PCR analysis for potentially positive or negative control by OxyR. Differences were found in 7 of 40 genes when comparing an *oxyR* mutant vs. PAO1 expression that was confirmed by ß-galactosidase reporter assays. Among these, *phnW*, encoding 2-aminoethylphosphonate:pyruvate aminotransferase, exhibited reduced expression in the *oxyR* mutant compared to wild-type bacteria. Electrophoretic mobility shift assays indicated binding of OxyR to the *phnW* promoter and DNase I footprinting analysis also revealed the sequences to which OxyR bound. Interestingly, a *phnW* mutant was more susceptible to *t*-butyl-hydroperoxide (*t*-BOOH) treatment than wild-type bacteria. Although we were unable to define the direct mechanism underlying this phenomenon, we believe that this may be due to a reduced efficiency for this strain to degrade *t*-BOOH relative to wild-type organisms because of modulation of AHP gene transcription in the *phnW* mutant.

## Introduction

*Pseudomonas aeruginosa* (*PA*) is an important human pathogen that is currently ranked fifth by the World Health Organization in overall infections [1]. Burn and cancer chemotherapy patients, chronic alcoholics, the immunocompromised (e.g., HIV infection) and patients suffering from cystic fibrosis (CF) and chronic obstructive pulmonary disease (COPD) lung disease are particular prone to highly problematic or fatal infections by this organism [2,3].

The response to such infections, especially in the airway, is mediated by macrophages and subsequently neutrophils, both of which are capable of triggering an oxygen-dependent antimicrobial respiratory burst. This burst involves the production of reactive oxygen intermediates (ROI) including superoxide (O_2_^-^), hydrogen peroxide (H_2_O_2_), the hydroxyl radical (HO^.^), peroxy acids and organic peroxides (e.g., lipid). The organism must also face production of reactive nitrogen intermediates (RNI), the most reactive being peroxynitrite (ONOO^-^), a product of nitric oxide (NO, generated by host NO synthases) and O_2_^-^. The defensive response to H_2_O_2_ and organic peroxides is governed by the global transactivator, OxyR [4]. *PA* OxyR triggers transcriptional activation of classical antioxidant genes encoding catalases A and B (KatA [5], KatB [6]), periplasmic alkyl hydroperoxide reductases B (AhpB) [6] and cytoplasmic AhpCF (6) but not **o**rganic **h**ydroperoxide **r**eductase, Ohr [7]. Ohr is specifically inducible by organic hydroperoxide but is not part of the OxyR regulon [7].

In this study, we first used a bioinformatics approach to identify genes controlled by OxyR based upon a canonical OxyR promoter recognition sequence ATAG-N_7_-CTAT-N_7_-ATAG-N_7_-CTAT. Of over 100 genes identified as being putatively under OxyR control, our goal was to identify those that would ***not*** be predicted to be involved in the classical oxidative stress response to identify the physiological breadth of the OxyR regulon. Here, we identified the *phnW* gene, encoding 2-aminoethylphosphonate:pyruvate aminotransferase, whose promoter region matched 12 of 16 bp of the aforementioned OxyR promoter recognition sequence. Surprisingly, PhnW was found to play a role in resistance to *t*-butyl hydroperoxide (*t*-BOOH), but not H_2_O_2_ nor cumene hydroperoxide (CHP). However, PhnW did play an indirect role in *t*-BOOH degradation *in vivo*, yet the purified protein did not appear to possess direct degradation or modification properties when exposed to *t*-BOOH *in vitro*.

## Materials and methods

### Bacterial strains, plasmids and planktonic growth conditions

The bacteria used in this study (**listed in** **Table 1**) were grown in Luria-Bertani broth (L-broth) supplemented with appropriate antibiotic(s) when required for selective pressure. Aerobic cultures were grown at 37°C with shaking at 200 rpm at a 1/10 volume to total Erlenmeyer flask ratio. Media were solidified with 1.5% Bacto-agar. Frozen bacterial stocks were stored at -80°C in a 1:1 mixture of 30% glycerol and stationary-phase bacterial suspension.

**Table 1:** Bacterial strains and plasmids used in this study.

Strains and plasmids	Genotype or description	Ref or source
*E*. *coli* DH5-αMC	F^-^ⱷ80d*lacZΔM15 endA1 recA1 hsdR17(r*_*K*_^*-*^ *m*_*K*_^*-*^*) supE44 thi-1 gyrA96 Δ(lacZYA-argF)*U169	Invitrogen
*oxyR*	*P*. *aeruginosa oxyR* mutant with removal of antibiotic resistance cassette	This study
*oxyR/*p*oxyR*	*P*. *aeruginosa oxyR* mutant containing pUCP20-*oxyR*	This study
*ohr ahpC*	*P*. *aeruginosa ohr* and *ahpC* double mutant	This study
pQF50	Broad-host-range *lacZ* transcriptional fusion, Cb^R^	[8]
pEX100T	Vector to construct mutants in *P*. *aeruginosa*, broad- host-range, Cb^R^	[9]
miniCTX	Site-specific integration-proficient plasmid for *P*. *aeruginosa*, Tc^R^	[9]
pUCP20	Pseudomonas shuttle vector, Cb^R^	[10]
PA *phnW*::Gm	*P*. *aeruginosa phnW* mutant containing Gm cassette	This study
pEX100T *phnW*::Gm	pEX100T with Gm integrated in *phnW*	This study
pQF50 *phnW-lacZ*	pQF50 with upstream sequence of *phnW* containing OxyR putative binding domain fusion with *lacZ*	This study
pUCP2*0 phnW*	pUCP20 containing complete sequence of *phnW* gene	This study
PA *phnW*::Gm/p*phnW*	*P*. *aeruginosa phnW* mutant containing pUCP2*0 phnW*	This study
*oxyR phnW*::Gm	*P*. *aeruginosa oxyR* and *phnW* double mutant	This study
*oxyR phnW*::Gm/p*phnW*	*P*. *aeruginosa oxyR* and *phnW* double mutant containing pUCP2*0 phnW*	This study
*ohr ahpC/*p*phnW*	*P*. *aeruginosa ohr* and *ahpC* double mutant containing pUCP2*0 phnW*	This study
SM10	*thi-1 thr leu tonA lacY supE recA*::RP4-2-Tc::Mu (Km^r^)	This study

### Manipulation of recombinant DNA and genetic techniques

Transcription of the *phnW* gene was studied by using pQF50-based promoter fusion assays [8]. A *phnW*::Gm mutant was constructed using the pEX100T allelic exchange system and 6% sucrose counter-selection [11]. Complementation of this mutant was performed using pUCP19, an *Escherichia*-*Pseudomonas* shuttle vector [9]. All techniques were performed based upon standard cloning protocols [9,11,12]. All PCR products were amplified using *pfu* DNA polymerase (BRL) using an MJ research thermal cycler and the nucleotide sequence confirmed using the Cincinnati Children’s Hospital Medical Center DNA sequencing and Genotyping Facility.

### Analysis of potential OxyR-regulated genes in *PA* PAO1

Potential OxyR-binding sites identified in the *PA* PAO1 genome were based on similarity to the well characterized *Escherichia coli* OxyR-regulated promoter sequences (ATAG-N_7_-CTAT-N_7_-ATAG-N_7_-CTAT) [6]. The DNA motif search tool in the Pseudomonas Genome Database was used to scan the PAO1 genome [12]. Candidate sites were further screened based on the type of genomic region that the site was found in intergenic versus putative open reading frames and whether proteins encoded by the downstream gene were predicted to be secreted past or embedded within the cytoplasmic membrane [12]. As predicted, representatives of the OxyR-regulated promoters under oxidizing conditions (e.g., H_2_O_2_, CHP, *t*-BOOH exposure) were the well-established *PA* OxyR-controlled genes *katB*, *ahpB* and *ahpCF* genes, respectively [6,13] (**Table 2**).

**Table 2:** The bioinformatic results of OxyR-dependent genes candidate and some well-known OxyR-dependent genes.

Gene Name	Gene Product	Matching bases	Motif Location	Distance to gene	Motif Sequence—separated into 4-mers						
					ATAG	Spacer	CTAT	Spacer	ATAG	Spacer	CTAT
PA3236	probable glycine betaine-binding protein precursor (BetX)	13	3624730–3624766	187	ATCG	GCCTGTC	CAAT	CGAGTGC	ATAG	AGCGCTT	CGAT
PA0848	probable alkyl hydroperoxide reductase (AhpB)	13	927016–927052	95	ATAG	GCTGACT	CTAT	CGTGCGA	ATTG	AAATTCG	ACAT
PA0846	probable sulfate uptake protein	13	924922–927147	2094	ATGT	CGAATTT	CAAT	TCGCACG	ATAG	AGTCAGC	CTAT
PA1310	2-aminoethylphosphonate:pyruvate aminotransferase (PhnW)	12	1420198–1420162	128	ATCG	GCCTGGC	TTAT	GGCTGGC	ATCG	GAACAAA	CAAT
PA0139	alkyl hydroperoxide reductase subunit C (AhpC)	12	158120–158084	101	ATAG	ATTTAGG	TAAT	CAGTGAA	ATGG	TCTAAAT	CAAT
PA2957	probable transcriptional regulator	10	3317470–3317506	32	ATCG	AAGCCGC	GTAT	TATGCCT	ATTC	AGCACGA	AAAT
PA1097	transcriptional regulator (FleQ)	10	1187514–1187478	73	ATAA	GCAGCCA	GCAT	TTGGCCA	CTAG	TTAAGTC	AAAT
PA1898	quorum-sensing control repressor (QscR)	10	2068974–2068938	516	ATCG	GCACGGA	CAAT	GAAATGC	CTGG	TCGAATT	AAAT
PA1423	probable chemotaxis transducer (BdlA)	10	1549778–1549814	191	ATAT	TTCCGAC	GAAT	GGCGGTA	ATTT	GTTTCAC	CCAT
PA5095	probable permease of ABC transporter	10	5738331–5738295	974	ATCT	GGAGCGT	CGAA	CCATGCG	ATAC	AAGAAGT	CGAT
PA4613	Catalase (KatB)	9	5171801–5171837	75	ATTG	AAAAACC	TAAT	CGCGCCG	GTGA	GGAATAT	CAAT
PA2999	Na+-translocating NADH:ubiquinone oxidoreductase subunit Nrq1 (NqrA)	9	3357556–3357592	57	ATCA	TCGCGCG	CAAC	GTACTGA	ATTG	GCACGAT	TGAT
PA1003	Transcriptional regulator (MvfR)	9	1087437–1087473	342	ATTC	GACGAAA	AGAA	AATCCGG	ATAT	TTACCGG	TTAT
PA0192	probable TonB-dependent receptor	9	218860–218824	312	ATGC	CTTTGTC	CAAT	ATTTCCA	ATGC	CGGTAAA	GCAT
PA1648	probable oxidoreductase	9	1795990–1796026	103	ATGA	TTCGCCG	CCAT	CAGTCTT	ATCA	GCGGGCT	GGAT
PA4403	secretion protein (SecA)	9	4937680–4937644	1028	ATGG	TGCCGGC	TCAG	AAAAATG	ATGT	GCATGAC	TTAT
PA3242	probable lauroyl acyltransferase	9	3630801–3630765	160	ATGA	GCCAAGC	CGAC	CTCCTCG	ATCA	AGACCCC	GTAT
PA0594	peptidyl-prolyl cis-trans isomerase (SurA)	9	654104–654068	296	ATAT	GGCAACG	AAAA	CGACATC	ATCA	AGCAGCA	CGAT

### Semiquantitative expression of OxyR-dependent genes by RT-PCR

Total RNA from exponential phase *PA* and *oxyR* mutant bacteria was purified using a RiboPure-Bacteria kit as specified by the manufacturer (Ambion). After treatment with DNase I, 1 μg of total RNA was converted to cDNA by using random primers and reverse transcriptase (Promega). Then, 1 μl of cDNA from either *PA* PAO1 or *oxyR* mutant bacteria was used as a template to amplify the genes of interest that were not of the classic antioxidant variety (e.g., catalase/peroxidase) as well as *omlA*, the latter of which is a constitutively expressed gene and is used frequently as a reliable, internal control [14] using *Taq* DNA polymerase. After designated cycles of the RT-PCR were completed, amplified products was analyzed by electrophoresis on 1.2% agarose gels after sampling every 5 cycles between 15–45 cycles at 55°C. Band intensities were determined by using AlphaEase FC StandAlone software (Alpha Innotech). Then, the optimum PCR cycles were used to amplify a 253-bp *phnW* fragment using both *PA* PAO1 and *oxyR* mutant bacteria, respectively.

### ß-galactosidase assays

All bacteria containing transcriptional *phnW*-*lacZ* fusions were grown in L-broth to exponential phase and exposed to 250 μM of *t*-BOOH for 30 min. Cell-free extracts from selected bacterial strains harboring the *phnW*-*lacZ* fusions were then used to assay ß-galactosidase activity [15]. All assays were performed at least in triplicate, and the values recorded as the mean +/- standard error.

### Electrophoretic mobility shift assay (EMSA)

A 199-bp DNA fragment upstream of the *phnW* gene containing the putative OxyR binding domain was amplified using *Pfu* DNA polymerase. The accuracy of this DNA fragment was confirmed by DNA sequencing analysis. The EMSA was performed following the DIG gel shift protocol, 2^nd^ generation (Roche). Briefly, the DNA fragment was 3’-end-labeled with terminal transferase and DIG-11-ddUTP. Then, 0.8 ng of DNA labeled probe was added to the binding reaction with increasing amounts of OxyR protein using bovine serum albumin as a competitor to demonstrate OxyR specific binding. The reaction mixture was then separated by electrophoresis on 6% non-denaturing polyacrylamide gels. The protein-DNA probe complex was transferred to positively charged nylon membranes by electroblotting followed by signal detection using chemiluminescence.

### DNase I footprinting analysis

A radioactively labeled DNA fragment harboring putative OxyR recognition sequences was prepared by PCR. The reaction was initiated using a PA1310FW primer (5´-CAGGCGCTCGGCGGCGCG-3´) and a PA1310RV primer (5´-CTTCGCGGTCCGGGCGGT-3´) generated a 199-bp DNA fragment to identify the OxyR binding motif upstream of the *phnW* gene. Labeled probe (50 pmol) was added to binding buffer [20 mM Tris-HCl, pH 7.0, 50 mM KCl, 1 mM EDTA, 5% glycerol, 50 μg ml^-1^ BSA, 5 μg ml^-1^ calf thymus DNA, 0.5 μg ml^-1^ poly-(dI-dC)]. After the addition of purified OxyR (500 and 1000 nM), the reaction was incubated at 25°C for 15 min. Subsequently, 25 μ l of 0.2 mM Mg^2+^-0.1 mM Ca^2+^-0.5 U of DNase I was added to the binding reaction, and incubated for an additional 1 min. Finally, 200 μl of stop solution (20 mM EDTA, pH 8.0, 1.0% SDS and 0.2 M NaCl) was added. The mixture was then extracted with phenol-chloroform and the DNA precipitated with 95% ethanol at -20°C. The pellets were resuspended in sequencing buffer and loaded onto a 5% denaturing polyacrylamide sequencing gel. After transfer of the sequencing gel to PVDF membranes, the OxyR-specific footprint was assessed by autoradiography.

### Filter paper disk assay for *t-*BOOH sensitivity

An exponential phase bacterial suspension was added to 5 ml of fresh L-broth containing 0.8% low-melting-point agarose (SeaPlaque) to a final O.D._600 nm_ of ~0.01. The suspensions were then distributed evenly on the LB agar surface. After the agarose solidified, filter paper disks (7 mm) impregnated with 10 μl of either 0.3 M or 0.5 M *t*-BOOH were placed on the top-agar surface and the plates were incubated at 37°C for 24 hr. The zones of killing were measured and the results are presented as the average from at least 3 independent experiments +/- standard error (SE).

### *t*-BOOH degradation assay

The ability of *PA* and selected isogenic mutants to degrade *t*-BOOH was investigated as previously described [16]. Briefly, overnight cultures of all *PA* bacteria (wild type, mutant or complemented strains) were inoculated into L-broth to a final O.D._600_ of 0.1 and allowed to grow at 37°C with shaking for ~3 hr, at which point various cells were diluted into fresh L-broth at an O.D._600_ of 0.4. Then, 200 μM of *t*-BOOH was added to each culture. The remaining amount of *t*-BOOH was determined at 0, 6, 9 and 12 min, respectively. At each time point, 1 ml of untreated and treated cells were collected by centrifugation to clarify the bacteria. An aliquot (100 μl) was added to 400 μl of 25 mM sulfuric acid followed by 500 μl of freshly prepared reaction buffer (200 μM ferrous ammonium sulfate, 200 μM xylenol orange, and 25 mM sulfuric acid). The reaction was incubated for 10 min at room temperature and the optical density of the solution measured at 540 nm. The concentration of remaining *t*-BOOH in the culture was calculated using a standard curve that was repeated for each set of experiments. To investigate the potential ability of purified PhnW to degrade or modify *t*-BOOH, PhnW (see below for purification details) was added to a solution containing 200 μM of *t*-BOOH and 100 μM of the reductant, dithiothreitol (DTT). Finally, the reaction mixture was incubated at 37°C and investigated for the degradation of *t*-BOOH as mentioned above at 15, 30 and 60 min, respectively.

### Purification of PhnW

The *phnW* coding region was amplified from *PA* genomic DNA using *Pfu* DNA polymerase with primer *phnW* F-pET *Bam*HI (5-ACTGGATCCATGAGCACTGCCGAACGCGCACCCAT-3´) and primer *phnW* R-pET *Not*I(5´-TATGCGGCCGCGATCTCGAGGACTTCCAGTTCGCGCAGC-3´). The expected PCR product which is ~1.1 kb was digested with *Bam*HI and *No*tI and ligated within compatible sites of the expression vector, pET23a, creating pET-*phnW*. *E*. *coli* BL21(DE3)/pLysE containing pET-*phnW* was grown in L-broth supplemented with 100 μg/ml ampicillin at 37°C with shaking until the O.D._600_ of the cells reached 0.6–0.8. Then, 1 mM IPTG was added and incubated for 3 hr to induce the expression of PhnW. The cell pellet was kept at -20°C until use. To initiate purification of this protein, the cell pellets were resuspended in sonication buffer (50 mM NaH_2_PO_4_, 20 mM Tris-HCl pH 8.0 and 100 mM NaCl and 20 mM imidazole). After sonication with a Sonic Dismembrator Model 500 for 15 min on ice, the supernatant was separated from cell debris by centrifugation at 13,000 x *g* for 30 min at 4°C. The supernatant was loaded on a Ni-NTA agarose column pre-equilibrated with sonication buffer. Non-specific binding proteins were removed with 10 column volumes (CVs) of washing buffer (sonication buffer with increasing imidazole concentration to 50 mM). PhnW was eluted from the Ni-NTA agarose column using 5 CVs of elution buffer (sonication buffer with increasing the imidazole concentrations to 100 mM). The fractions containing the PhnW protein were pooled and dialyzed against 50 mM Tris, pH 8.0, 5% glycerol, 0.05 M NaCl and 0.1 mM EDTA.

### Use of KEGG (Kyoto Encyclodedia of Genes and Genomes) pathway database for identification of the potential role of PhnW in cellular reducing power

The KEGG Pathway Database (http://www.genome.jp/kegg/pathway.html) was used to identify where PhnW lies in fundamental *PA* metabolism. First, after clicking on the “Organism” bar, we typed in “Pseudomonas” and scrolled down to *PA* strain PAO1 (codename “pae”). We then scrolled down to section 1.6 (Metabolism of other amino acids) and selected subsection 00440 for “phosphonate and phosphinate metabolism.” The *phnW* gene is coined PA1310 in the *PA* PAO1 genome (www.pseudomonas.com) and we subsequently entered *phnW* in the open box and hit return. An elegant pathway map is given with the PhnW enzymatic reaction presented in red font with the enzyme commission number of 2.6.1.37 in **S2 Fig**.

## Results

### Analysis of putative *PA* genes under OxyR control using a bioinformatics analysis

The entire *PA* PAO1 genome was first screened for OxyR-binding domains using the OxyR DNA recognition sequence ATAG-N_7_-CTAT-N_7_-ATAG-N_7_-CTAT [6]. The results are listed in **Table 2**. The bases listed are those that are conserved in the OxyR promoter sequences for the experimentally confirmed *katB*, *ahpB* and *ahpCF* that we published 16 years ago [6] and more recently using ChIP-chip analyses which also includes the *bdlA* (**b**iofilm **d**ispersion **l**ocus, [17]) and PA3236 genes [18]. We were particularly interested in the promoter regions of potentially OxyR-regulated genes that were ***not*** predicted to be involved in the classical response to oxidative stress (such as genes encoding catalases A [5] and B [6], alkyl hydroperoxide reductases (Ahps, [6]), organic hydroperoxide reductase (Ohr), overlapping regulon genes (Ssp), and membrane proteins involved is the Ssp response. The optimal score for the OxyR box analysis was 13/16, for the upstream regions of the *betX* and *ahpB* genes, the latter of which is periplasmic, known to be controlled by OxyR, and involved in protection against both H_2_O_2_ and alkyl hydroperoxides [6]. The promoters of two other known OxyR-controlled genes included the *katB-ankB* operon and *ahpCF* that received scores of 9/16 and 12/16, respectively. In contrast to a recent study by Wei *et*. *al*. [19] who identified genes under OxyR control when organisms were exposed to H_2_O_2_, we found that only 8/40 of the genes matched from bioinformatic studies in the aforementioned study. These genes were *betX*, *ahpB*, *ahpC*, *bdlA*, *katB*, *nadA*, *mscL* and *mvfR*, respectively.

### Semi–quantitative expression of genes culled from bioinfomatic predictions by reverse transcriptase polymerase chain reaction (RT-PCR)

**Fig 1:**
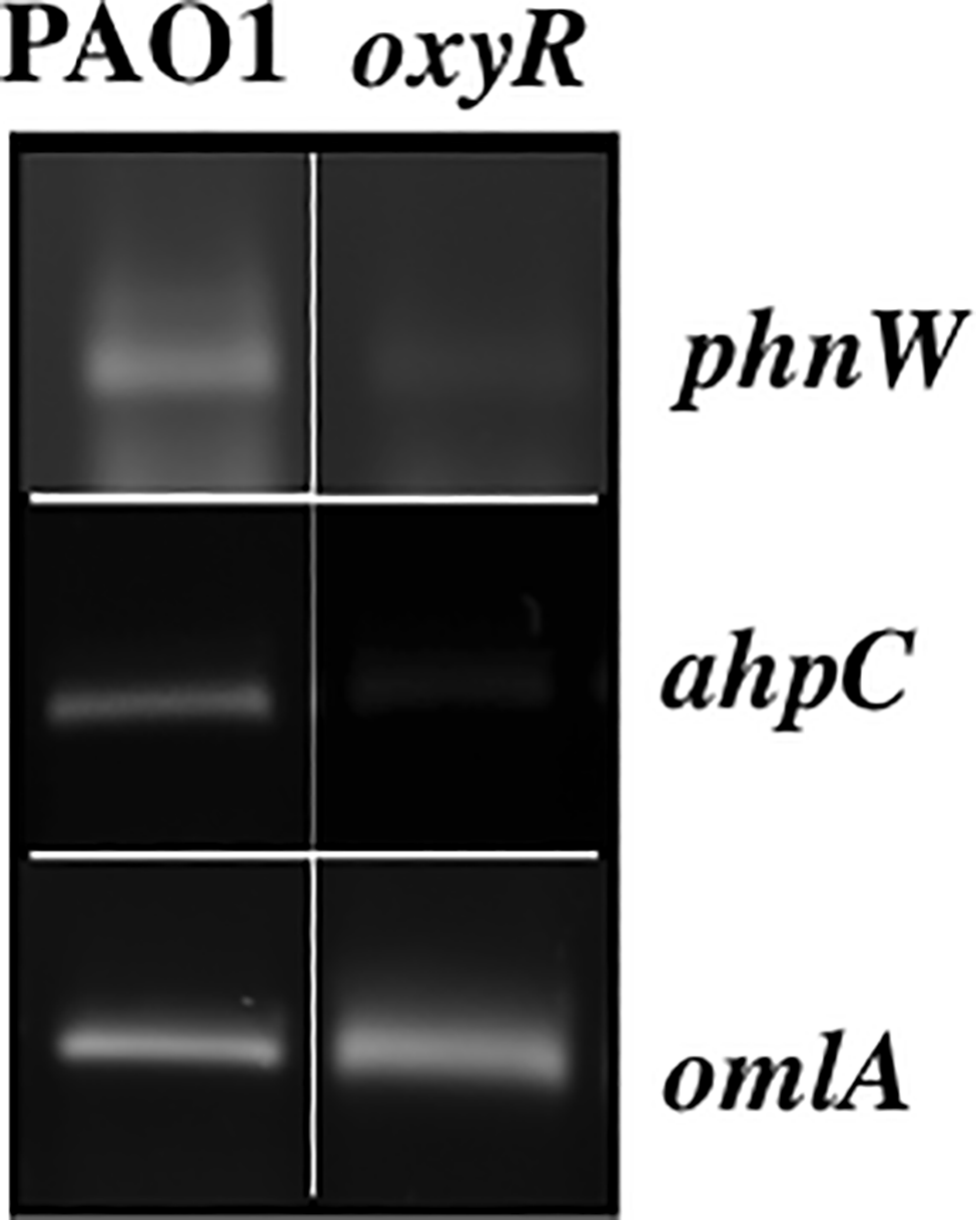
Semi-quantitative expression of PA *phnW*. Total RNA was isolated from exponential phase *PA* PAO1 or its isogenic *oxyR* mutant. Then, 1 μl of cDNA was used to amplify the *phnW* promoter region with specific primers. The *omlA* gene was used as an internal constitutive control [14] and *ahpC* was used as a positive gene under OxyR control [6].

**Fig 2:**
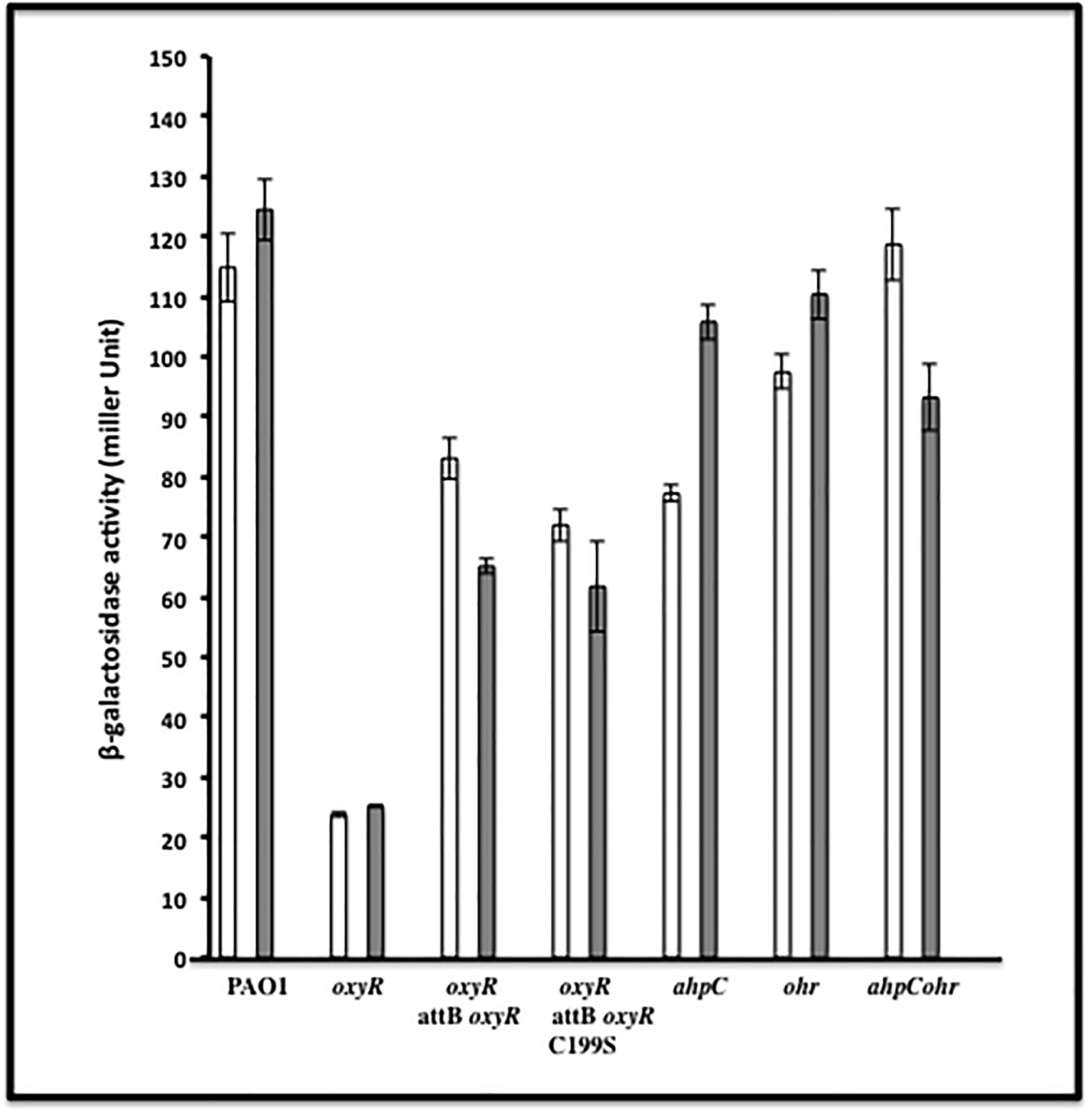
Determination of *phnW* expression levels in wild-type and mutant strains when exposed to *t*-BOOH. All bacteria which contained a *phnW*-*lacZ* transcriptional fusion plasmid (pQF-*phnW*) were grown to exponential phase and exposed to 250 μM *t*-BOOH. ß-galactosidase activity assays were reported as the mean +/- standard error compared to untreated bacteria. The assays were performed using three independent experiments. The white bars represent untreated bacteria while the gray bars represent *t*-BOOH treated organisms, respectively.

Our first experiment to corroborate our bioinformatic predictions that OxyR either positively or negatively controls transcription of specific genes was by performing RT-PCR from exponential phase bacteria. Genes that matched 9–13 of 16 nucleotides of the OxyR binding motif used in our bioinformatic search that did not encode predicted hypothetical proteins (totaling 40 genes), were amplified and compared between an *oxyR* mutant and wild-type bacteria. Our results revealed that 3/40 genes could not be amplified (even with 1 μg of RNA). Duplicate results of the RT-PCR analysis revealed differences in gene expression of 15 genes compared between wild-type bacteria and the *oxyR* mutant. These included *mvfR*, *qscR*, PA2957, PA0846, *betX*, PA0192, PA1097, PA1423, *phnW*, PA1648, PA3242, PA4403, PA2999, PA0594 and PA5095 (**Table 2**). Among this group, the RT-PCR results of the *phnW* gene, encoding 2-aminoethylphosphonate:pyruvate aminotransferase, revealed lower gene expression levels in the *oxyR* mutant when compared to wild-type bacteria under non-inducing conditions (**Fig 1)**, which was also confirmed transcriptionally by ß-galactosidase activity assays (**Fig 2)**. The upstream sequence that is considered a putative OxyR binding domain of *phnW* matched 12 of 16 bases. Two sets of control genes were used; the *omlA* gene which is a constitutively expressed internal control [14], and a positive control gene, *ahpC*, which is a known member of the *PA* OxyR regulon [6].

### Confirmation of OxyR regulation of *phnW* by transcriptional ß-galactosidase reporter fusion analyses

From our previous experiments using RT-PCR, only 4/15 genes (*phnW*, *bdlA*, *bexT* and *qscR*) that showed lower expression in the *oxyR* mutant were further evaluated by transcriptional ß-galactosidase reporter assays. Herein, we focused only on the *phnW* gene. The upstream region of the *phnW* gene which contains the OxyR binding domain was cloned into pQF50, a transcriptional fusion vector [8], and then transformed into the following strains: PAO1, *oxyR*, *oxyR-attB oxyR*, *oxyR-attB oxyR* C199S, as well as *ahpC*, *ohr* or *ahpC ohr* double mutant bacteria. As a reminder, AhpCF and Ohr in *PA* are two of the major proteins involved in the cytoplasmic detoxification of organic hydroperoxides [6,7,13]. The results of the ß-galactosidase activity assays confirmed our RT-PCR results that *phnW* gene expression is lower in the *oxyR* mutant than in wild-type bacteria (**Figs 1 and 2)**. Our results also showed complementation of the *phnW* mutant in both cell backgrounds harboring either OxyR or OxyR C199S (constitutively OxyR reduced form), the latter of which is unable to sense oxidative stress [5]. Expression of *phnW* was also ~30% lower in the *ahpC* mutant while it was 80% lower in the *oxyR* mutant when compared to wild-type levels. Still, *phnW* expression could be induced to approximately parental levels by exposure to 250 μM of *t*-BOOH, but not in *ohr* or *ahpC ohr* mutant bacteria (**Fig 2**). However, exposure of *ahpC ohr* mutant bacteria to 250 μM *t*-BOOH caused a slight reduction (less than 10%) in *phnW* expression (**Fig 2**).

### Electrophoretic mobility shift assay (EMSA) of the *phnW* promoter region

**Fig 3:**
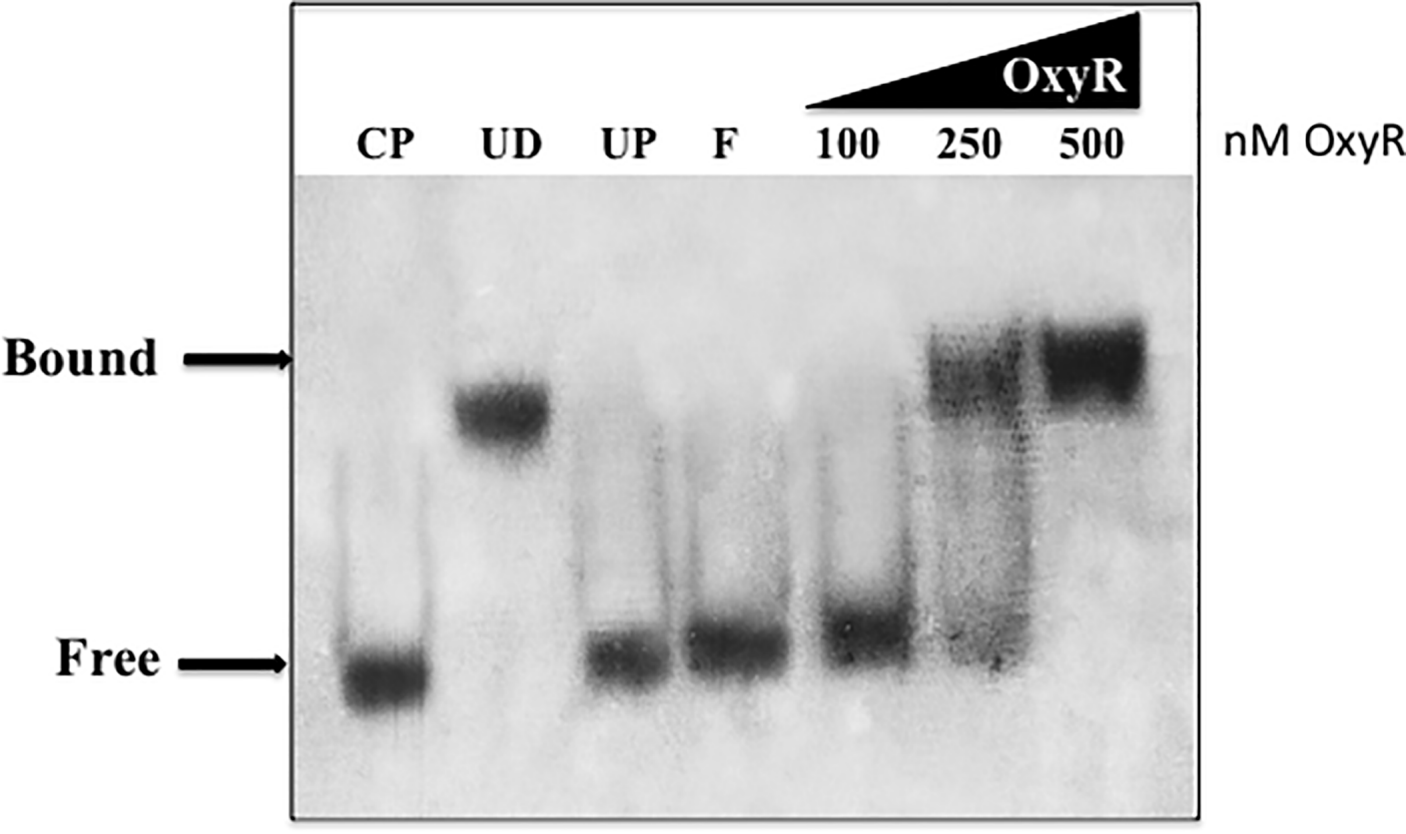
Electrophoretic mobility shift assay (EMSA) to indicate OxyR binding to the promoter region upstream of *phnW*. Purified OxyR was added to 0.8 ng of DIG-nonradioactive labeled 199-bp DNA fragment of *phnW* containing the putative *phnW* OxyR binding domain in the binding buffer and separated on a polyacrylamide gel as described in the materials methods section. The binding reaction consisted of a labeled *phnW* fragment and various quantities of OxyR protein. UP is the addition of 2 μg of **u**nrelated **p**rotein (BSA) to the binding reaction; F is **f**ree probe; the addition of increasing concentrations of OxyR (100, 250, 500 nM) to labeled *phnW* probe are listed; CP is the addition of 125-fold excess of unlabeled *phnW* DNA to the binding reaction; UD, the addition of 125-fold excess of **u**nrelated **D**NA (pUCP20 plasmid) to the binding reaction. The positions of free and bound *phnW* probe are shown on the left (see arrows).

Next, we assessed the binding of purified OxyR to a 199-bp DNA fragment upstream of the *phnW* gene that contained the putative OxyR binding domain by the EMSA. First, OxyR binding to this fragment was abolished by an unlabeled *phnW* competing DNA fragment (**see CP in** **Fig 3**). To investigate the specific binding of OxyR on the putative OxyR-binding site upstream of *phnW*, the unrelated protein (BSA) or unrelated DNA (pUCP20) was added to the reaction. Our results showed no change in the mobility shift between the *phnW* fragment and OxyR protein (**see UP and UD in** **Fig 3, respectively**). These data revealed the specific binding of OxyR on the putative OxyR binding sequence upstream of the *phnW* gene.

### DNase I footprinting analysis of the *phnW* promoter

**Fig 4:**
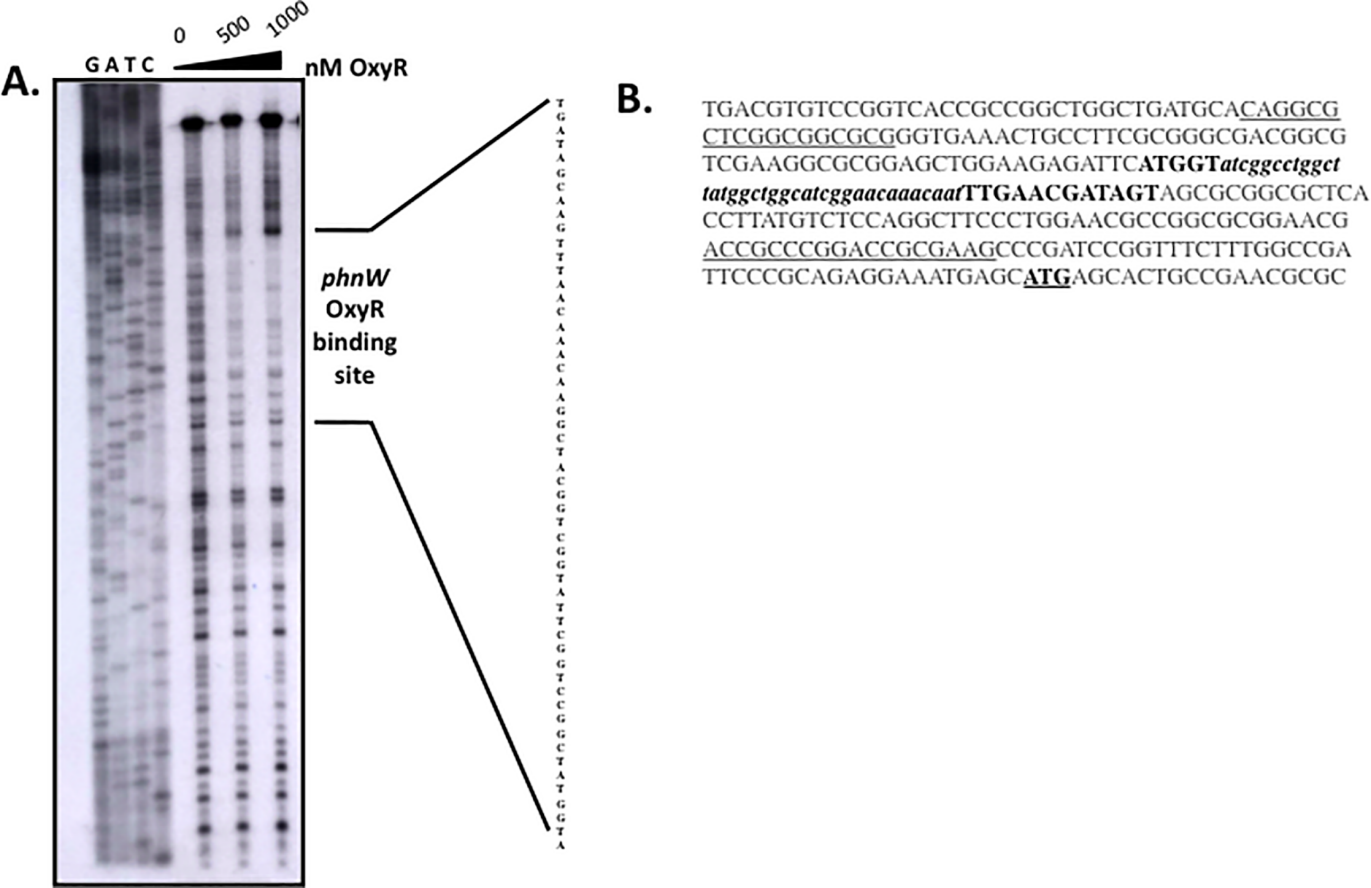
Localization of the OxyR binding domain of the *phnW* promoter by DNase I footprinting analysis. **(****A).** The 199-bp upstream sequence of *phnW* containing a putative OxyR domain was used to treat with DNase I in the presence of OxyR at 0, 500 and 1000 nM. The digested DNA was analyzed on a 5% denaturing polyacrylamide gel, followed by autoradiography. The sequence to which OxyR binds on this fragment was identified. (**B).** The upstream sequences of *phnW* that are underlined are the primers used in this study. The bold letters indicate the OxyR binding domain within the *phnW* upstream sequence while the lower case bold letters indicate the putative OxyR binding site based upon a match to the consensus ATAG-N_7_-CTAT-N_7_-ATAG-N_7_-CTAT sequence used to search for OxyR-dependent gene candidates. The underlined and bold ATG indicates the translational initiation codon of the *phnW* gene.

We next determined the precise location of the OxyR binding domain within the *phnW* promoter that was based upon our bioinformatic search using a DNase I footprinting analysis. Various concentrations of purified OxyR protein were used. The footprint pattern showed that OxyR at 500 nM binds to these upstream sequences (**Fig 4A)**. This putative OxyR binding domain is shown in bold, italic, and lower case nucleotide sequences in **Fig 4B**. We also performed a computer-based promoter prediction derived from 500-bp upstream of the *phnW* gene using the BDGP: Neural Network Promoter Prediction program (prokaryotic mode, reverse strand “no” command) and discovered a weak promoter score of 0.49; a perfect promoter score for this particular program is 1.0 (http://www.fruitfly.org/seq_tools/promoter.html). Such a low score indicated that the *phnW* gene is not a prioritized gene from the overall regulatory scheme in *PA*.

### Sensitivity of a *PA phnW* mutant to *t*-BOOH and partial rescue of *t*-BOOH resistance in *ohr ahpC* mutant bacteria

**Fig 5:**
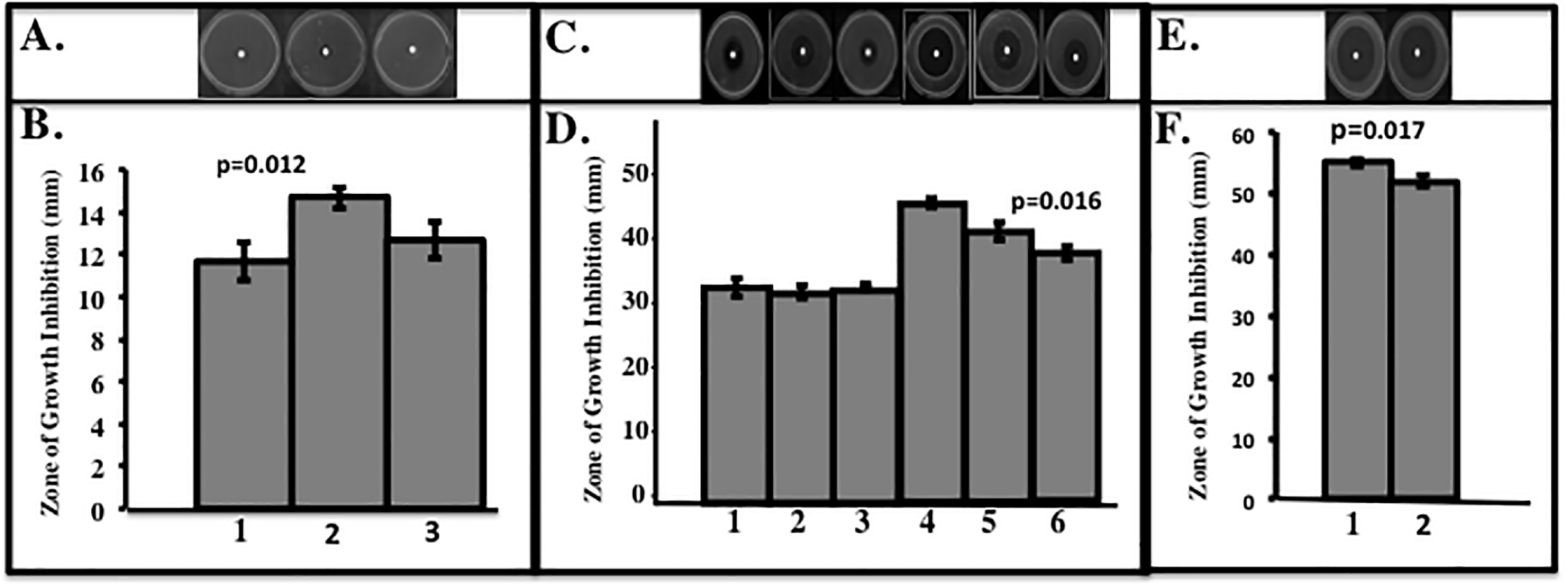
Sensitivity of bacteria to *t*-BOOH. Bacteria from the aerobic exponential phase (**A,B).** PAO1, *phnW*::Gm and *phnW*::Gm/p*phnW*) or (**E,F).** *ohr ahpC* and *ohr ahpC*/p*phnW*) or stationary phase (**C,D**). PAO1, *oxyR*/p*oxyR*, *phnW*::Gm, *oxyR phnW*::Gm, *oxyR* and *oxyR*/p*phnW*) were used to determine sensitivity to *t*-BOOH at 0.3 M (exponential) or 0.5 M (stationary), respectively. The experiments were independent and repeated at least three times. The values shown are means +/- standard error.

Sensitivity to *t*-BOOH of the isogenic *phnW* mutant relative to a number of mutants, complemented and wild-type strains was next examined. Our preliminary results indicated that the *phnW* mutant is only susceptibility to *t*-BOOH, and not to CHP and H_2_O_2_, respectively. Our results showed that the *phnW* mutant (**lane 2 vs. lane 1 in** **Fig 5A and 5B**) was more susceptible to *t*-BOOH compared to wild-type and complemented bacteria (**lane 3 vs. lane 1 in** **Fig 5A and 5B**) in the exponential but not the stationary growth phase (**lane 1 vs. lane 3 in** **Fig 5C and 5D**). In contrast, using stationary phase organisms, the *oxyR phnW* mutant was slightly more sensitive to *t*-BOOH than the *oxyR* mutant (**lane 4 vs. lane 5 in** **Fig 5C and 5D**), while provision of the *phnW* gene to the *oxyR* mutant provided some measure of protection against *t*-BOOH (**lane 6 vs. lane 4 in** **Fig 5C and 5D**). Moreover, provision of the *phnW* gene *in trans* could also protect a strain lacking AhpC and Ohr proteins (*ohr ahpC* mutant), that have previously been shown to be highly susceptible to organic hydroperoxides including *t*-BOOH [7]. Collectively, our results show that PhnW helps to partially protect the *ohr ahpC* double mutant from *t*-BOOH killing (**lane 2 vs. lane 1 in** **Fig 5E and 5F).**

### PhnW influences the cellular degradation rate of *t*-BOOH

**Fig 6:**
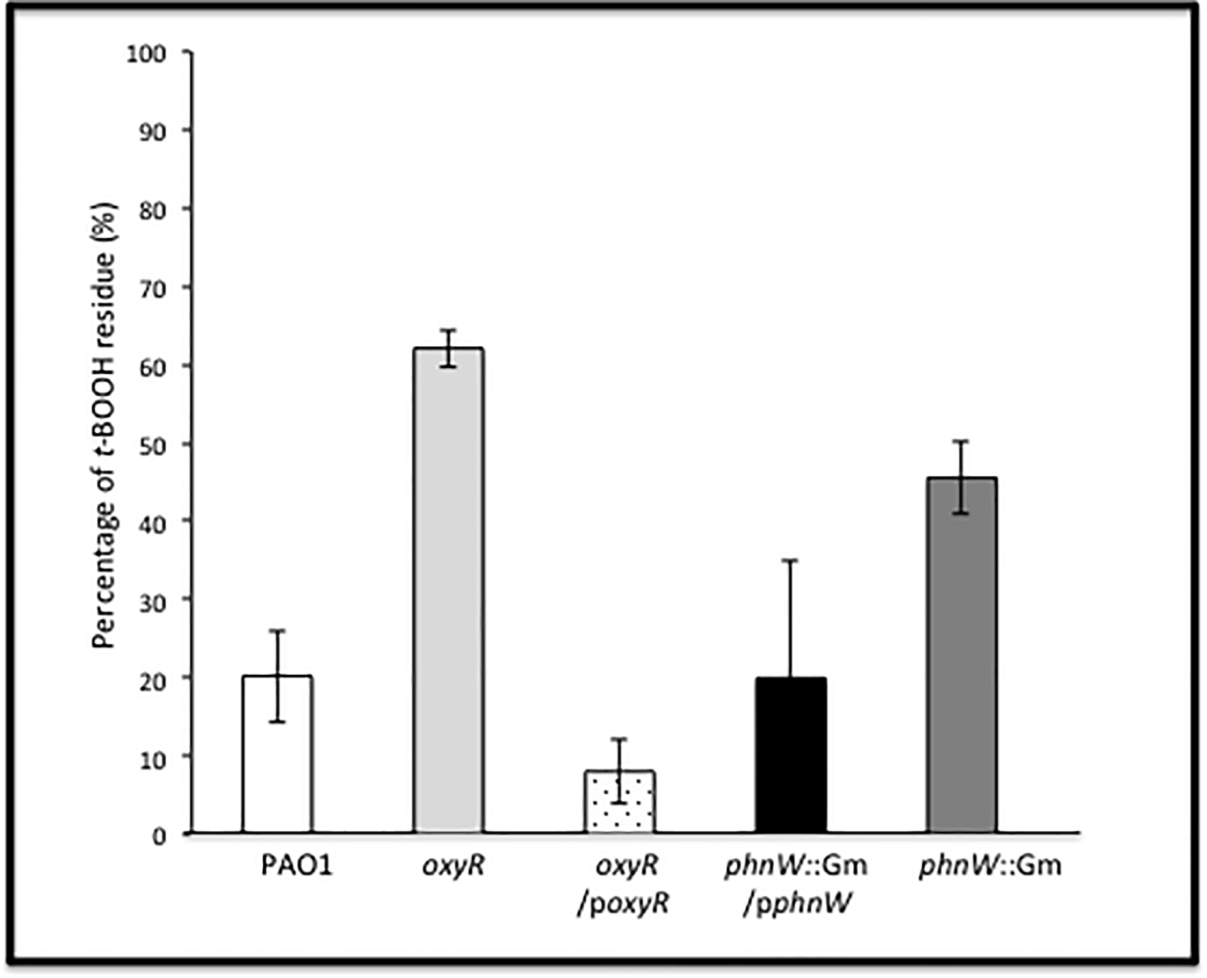
Time course of *t*-BOOH degradation by various *PA* strains. The rate of PA *t*-BOOH degradation, an organic hydroperoxide, was investigated using xylenol orange–iron reaction system as described in the materials and methods section. The exponential phase of PAO1 (white bar), *oxyR* (light gray bar), *oxyR*/p*oxyR* (dotted bar), *phnW*::Gm/p*phnW* (black bar) and *phnW*::Gm (dark gray bar) cells were used in this study in the present of 200 μM of *t*-BOOH and measured the rate of degradation of *t*-BOOH in each cell at difference time points. The percentage of *t*-BOOH remaining was reported after 12 min of incubation. The experiments were independently repeated at least three times and typical results are shown.

To determine whether PhnW influences the degradation rate of *t*-BOOH, a xylenol orange–iron reaction assay that allows for precise quantification of organic hydroperoxides was employed [20]. The rate of *t*-BOOH (200 μM) degradation by exponential phase, aerobic bacteria was measured in selected bacteria and the results were expressed as the percent of *t*-BOOH remaining in the media after 12 min incubation (**Fig 6)**. Wild-type bacteria (**white bar)** rapidly degraded *t*-BOOH, as did an *oxyR* mutant containing pUCP-*oxyR* (**white bar with dots**) or a *phnW* mutant containing pUCP-*phnW* (**dark** **gray bar**). As expected, the *oxyR* mutant revealed the slowest *t*-BOOH degradation rate (**light gray bar**) compared with all test strains. At *t*-BOOH exposure times from 0 to 9 min, the *phnW* mutant showed no difference in the rate of *t*-BOOH degradation compared to that of *oxyR*/p*oxyR*, *phnW*::Gm/p*phnW* and wild-type bacteria, but clear differences were evident in *t*-BOOH degradation at about 12 min after exposure (**Fig 6)**. The *phnW* mutant degraded only 55% of the *t*-BOOH compared to the 80% degradation observed in wild-type bacteria at the end of assay (12 min). The ability of purified PhnW to degrade *t-*BOOH also investigated. However, Purified PhnW did not show a direct ability to degrade *t*-BOOH *in vitro*.

## Discussion

The major response of *PA* and other Gram-negative bacteria including *E*. *coli*, *S*. *typhimurium* and *PA* to H_2_O_2_ and alkyl hydroperoxides is governed by the major LysR family member and trans-activator, OxyR [6,20,21,22]. The typical oxidative activation mechanism of OxyR requires formation of an inter-molecular disulfide bridge between amino acid residues 199 and 208 mediated by H_2_O_2_ or organic hydroperoxides [5]. Still, another new mechanism of OxyR-mediated gene activation anaerobic *S*-nitrosylation in *E*. *coli*, events that are distinct from OxyR-mediated gene activation under oxidative stess conditions [23]. However, the main dominant paradigm for bacterial responses to the aforementioned oxidants involves traditional antioxidant enzymatic machinery that includes but are not limited to catalases, peroxidases and alkyl hydroperoxide reductases. Other forms of OxyR-mediated gene regulation include the activation of regulatory RNAs including OxyS [24]. We have studied the role of *PA* OxyR and various antioxidants under its control for more than 17 years [5,6,17,22]. However, in this work, we wished to identify *PA* OxyR-dependent genes that would ***not*** be predicted to play a classical role in the oxidative stress response. First, we used the DNA motif ATAG-N_7_-CTAT-N_7_-ATAG-N_7_-CTAT that is the *E*. *coli* OxyR consensus binding sequence to search the *PA* genome database for genes that are possibly regulated by *PA* OxyR. When compared to a previous study by Wei *et al*., [18], who identified genes under OxyR control when organisms were exposed to H_2_O_2_, we found that only 8 of the 41 genes listed in **Table 2** matched those identified in this study. These genes were *betX*, *ahpB*, *ahpC*, *bdlA*, *katB*, *nadA*, *mscL* and *mvfR*, respectively. Among this gene list, *phnW* was not found by Wei *et al*., [17] to be under OxyR control and there was also no evidence or prediction by either manual or computer-based programs for any other protein to bind to the upstream sequences of the *phnW* gene [12].

The *phnW* gene, encoding 2-aminoethylphosphonate:pyruvate aminotransferase, can be found in the genomes of both pathogenic and non-pathogenic bacteria [25,26], yet its gene product is perceived to be involved in pathogenesis through metabolism of organophosphates. Surprisingly, its *phn*-based genes are all down-regulated (some several hundred-fold) when *PA* was grown in blood from burn patients, a phenomenon that is not understood at the mechanistic level [27]. *PA* PhnW catalyzes the transfer of the amino group of 2-aminoethylphophonate (ciliatine) to pyruvate, resulting in the production of 2-phosphonoacetaldehyde and alanine [28]. In this work, we found that the *phnW* gene was constitutively expressed, independent of whether OxyR was in its reduced or oxidized states, yet its expression was reduced 80% in an *oxyR* mutant. In contrast, *phnW* expression was found to be induced 30% in *ahpC* mutants relative to wild type bacteria (**Fig 2**). The significantly lower *phnW* expression in the *oxyR* mutant indicates that OxyR is a regulator of the *phnW* gene (**Figs 1 and 2**). This was confirmed by the ability of OxyR to bind specifically to *phnW* upstream sequences using both EMSA and DNase I footprinting analyses **(Figs 2 and 3).** *PA* OxyR contains the four putative OxyR binding tetranucleotide sequences with 67% identity to similar sequences in *E*. *coli* [29], results that are in agreement with our algorithm predictions. Previous EMSA studies by Wei *et al*. showed that as little as 84 nM OxyR can bind to the promoter region upstream of the *ahpC* gene [19]. This is in contrast to the requirement of ~3-fold higher levels of OxyR to bind to the binding domain upstream of the *phnW* gene. This result was also confirmed by our DNase I footprinting results, indicating that very high concentration of OxyR (500 nM) are needed for binding. A previous study by Ochsner *et*. *al*. [6] using EMSA indicated that 100 nM OxyR can bind to the well known OxyR-regulated gene promoters *katB*, *ahpB* or *ahpC*. In addition, DNase I footprinting of *E*. *coli* OxyR on the *ahpC* promoter was studied by Zheng *et*. *al*. [30], who showed that as little as 18 nM OxyR was required for promoter binding. This is about 28-fold or 2.5-fold less OxyR than we used in our DNase I footprinting or EMSA experiments, respectively. These two experiments clearly indicated a far lower binding affinity of OxyR to the *phnW* upstream nucleotides compared to the major OxyR-dependent antioxidant gene, *ahpC* [6], which is consistent with a very low promoter prediction score (0.49 of a possible perfect score of 1.0). This may result in dramatic differences in the potential for OxyR to control *phnW* expression in *PA*. Moreover, it is also supported by the alignment of the putative OxyR binding sequence of *phnW* with other OxyR-dependent genes base on the results of Wei *et al*., [17]. Our results indicate that the OxyR binding sequence upstream of *phnW* is 80% identical to *betX* (PA3236), 53% to the *ahpB*, *katB* and *ahpC* genes and 46% to *bdlA*, respectively (**S1 Fig).**

The *ahpB*, *katB* and *ahpC* are well known OxyR-dependent genes while *betX* and *bdlA* were both introduced to the literature by Wei *et*. *al*., [6]. Our results show that PhnW has an ability *in vivo* to partially protect bacteria from *t*-BOOH toxicity in *oxyR* and *ahpC ohr* mutants, two *t*-BOOH sensitive strains, and also in exponential phase but not stationary phase wild-type bacteria. Complementation of the lower expression of *phnW* in the *oxyR* mutant can occur using both *oxyR* and *oxyR* C199S, respectively. Interestingly under normal conditions, *phnW* expression is also lower when the bacteria lack only one of the major proteins involved in organic hydroperoxide detoxification (AhpCF or Ohr), while an absence of both AhpCF and Ohr allow for wild-type *phnW* expression. This may be a compensatory strategy triggered by PhnW for organisms to protect themselves from *t*-BOOH toxicity.

### How could PhnW contribute metabolically to the protection of *PA* from *t*-BOOH?

Because PhnW offered protection against *t*-BOOH *in vivo*, we sought in earnest to mechanistically define how this might occur. The forward substrate of PhnW, 2-aminoethylphosphonate, is taken into the cell by an ABC transporter system and can be utilized as a sole carbon source for growth [28]. Still, despite the fact that many enzymes can possess dual functions (e.g., catalase/peroxidase), we thought it highly doubtful that PhnW itself possessed any *t*-BOOH degrading activity and we subsequently proved this experimentally. Rather, we posited that L-alanine, one product of PhnW, could modulate cellular antioxidant levels. L-alanine has been shown to trigger activation of an antioxidant response in eukaryotes [31]. Alternatively, alanine can funnels into pyruvate that subsequently enters the TCA cycle, resulting in the net production of 4 NADH and one FADH_2_ molecule per pyruvate molecule. The significance of an increase in reducing power (electrons) is that many alkylhydroperoxide reductases such as the *E*. *coli* AhpCF require NADH as a bound cofactor [32]. Another possibility is that the reverse reaction of PhnW leads to the production of acetate and acetaldehyde (**see panel B inS2 Fig**, **black arrows on upper left**) which can be funneled into the glycolytic pathway, leading to more reducing power starting with the two NADH molecules produced from pyruvate dehydrogenase and the following six NADH and 2 FADH_2_ molecules from the TCA cycle. The substrate of PhnW protein (aminoethylphosphonic acid) was also investigated its ability to help protect bacteria from *t*-BOOH toxicity. Our results demonstrated that supplementation of aminoethylphosphonic acid in the media slightly helped to protect bacteria from *t*-BOOH toxicity at concentrations as low as 1 mM or as high as 10 mM. Surprisingly, the protection afforded by bacteria grown in the presence of aminoethylphosphonic acid was also specific to the *ahpC ohr* double mutant only. The killing zone with 0.2 M *t*-BOOH of stationary phase an *ahpC ohr* double mutant was 42±0.063 mm and 40±0.031 mm for either control bacteria or organisms supplemented with 10 mM aminoethylphosphonic acid, respectively.

**Fig 7:**
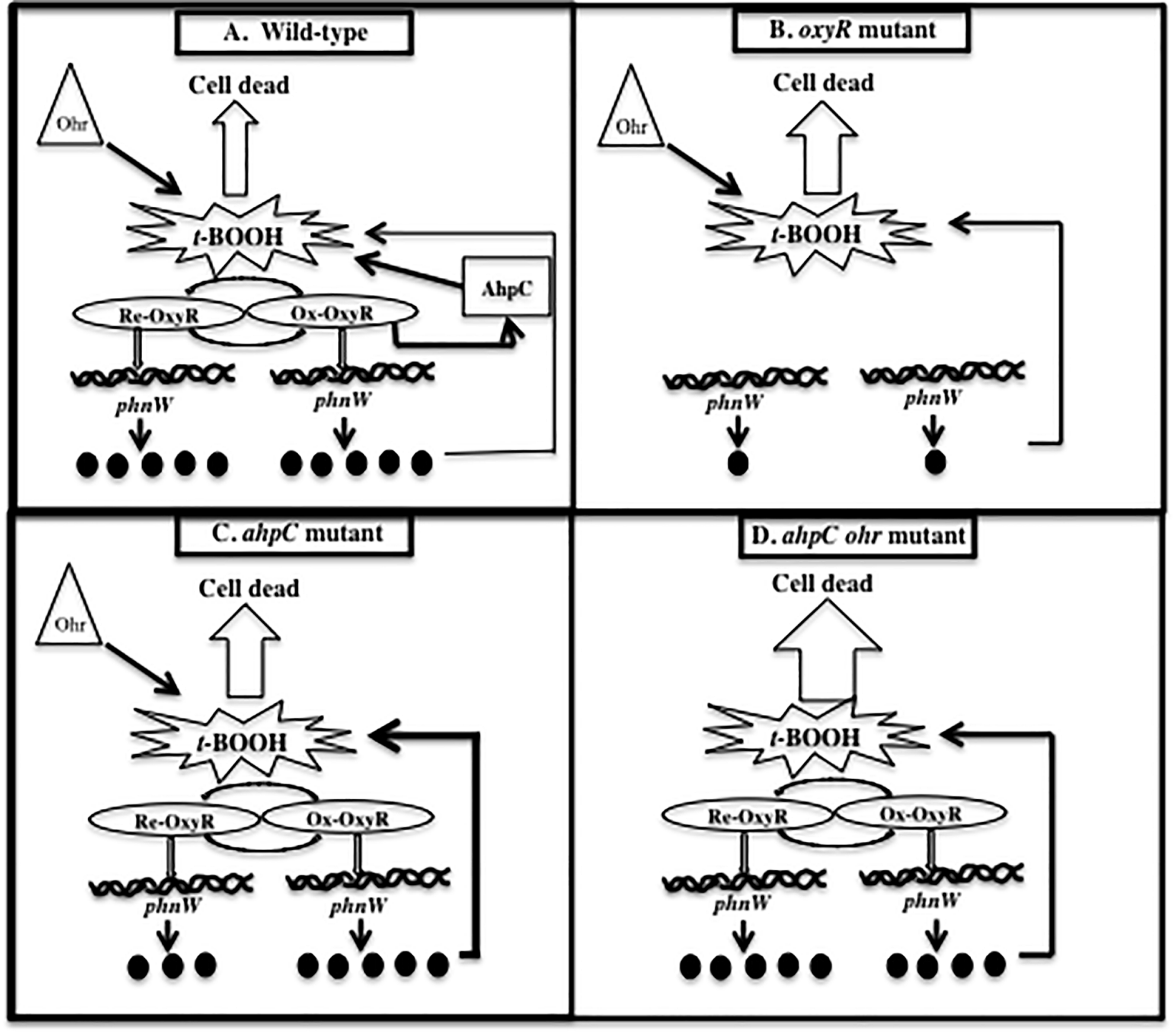
A model of *phnW* expression under difference conditions. **(A).** In wild-type bacteria, *phnW* is continuously expressed in both untreated bacteria and during exposure to *t*-BOOH, while oxidized OxyR triggers activation of *ahpC* expression as previously shown [6]. Wild-type bacteria used both of these proteins and Ohr to assist in either the direct or indirect degradation of *t*-BOOH. This results in efficient *t*-BOOH degradation to a by-product that is not toxic to the bacteria, thereby preventing damage to proteins, DNA and lipid. (**B).** Expression of the *phnW* gene is significant lowered in *oxyR* mutant bacteria (80%) when compared to wild-type bacteria with no induction of *ahpC* expression when exposed to *t*-BOOH. This results in greater susceptibility to *t*-BOOH of this mutant relative to wild-type bacteria since this strain likely only uses Ohr to help detoxify *t*-BOOH. (**C).** Lower expression of *phnW* was also detected in a PAO1 *ahpC* mutant compared to wild type bacteria (30%). A lack of this major AHP in response to *t*-BOOH coupled with lower expression of *phnW* with only Ohr detoxifying power remaining results in less protection of this mutant from *t*-BOOH toxicity. (**D).** An *ahpC ohr* mutant showed a slightly reduced expression of *phnW* when bacteria were exposed to *t*-BOOH, but no difference were observed under control conditions when compared to wild-type expression. Thus, a lack of both of the major *t*-BOOH detoxification proteins and a lower expression of *phnW* results in this mutant being the most susceptible to *t*-BOOH. This may aid in bacterial protection from endogenous free radicals that are continuously generated under aerobic conditions and/or at the earliest time period, when exposed to *t*-BOOH while the responding gene is not yet expressed. When the bacteria are exposed to *t*-BOOH, oxidized OxyR governs over-expression of AhpCF to help in its detoxification and together with Ohr, an OxyR independent *t*-BOOH detoxification protein (**Fig 7A**). A significantly lower expression level of *phnW* gene (~80%) was revealed in the *oxyR* mutant under both reduced and oxidized conditions, indicative of OxyR-mediated regulation of the *phnW* gene (**Figs 2 and 1**). When the *oxyR* mutant that had significantly reduced expression of AhpC and PhnW was exposed to *t*-BOOH, this event triggered an increased susceptibility to this oxidant when compared to wild-type bacteria (**Fig 7B**). Interestingly, *phnW* expression levels were also ~30% lower when compared to wild-type levels but are complemented when exposed to *t*-BOOH in the *ahpC* but not in the *ohr* mutant (**Fig 2**). This could be yet another mechanism by which bacteria used to protect themselves from *t*-BOOH toxicity when they lack the major *t*-BOOH detoxifying protein, AhpC (**Fig 7C**). Both *PA oxyR* and *PA ahpC* mutants still have an Ohr (organic hydroperoxide resistance), one of the major proteins that can contribute to *t*-BOOH detoxification. A genome search for “peroxidase” and “hydroperoxide” revealed 6 and 5 hits, respectively. This indicates that there are likely multiple redundant mechanisms to dispose of hyperoxides such as *t*-BOOH. Therefore, we expected that *phnW* expression should be higher in the *ohr ahpC* double mutant to protect bacteria cell from *t*-BOOH. Surprisingly, expression was ~20% lower in this strain after exposure to *t*-BOOH. The lower expression of PhnW after exposure to *t*-BOOH in the *ohr ahpC* double mutant may also contribute to the sensitivity of this double mutant to *t*-BOOH (**Fig 7D**). AhpC is OxyR-dependenct but Ohr is not. Therefore, it appears that *phnW* expression levels depend on the present of OxyR in the cell and also the level of AhpC in *ahpC* or *ahpC ohr* mutants. Therefore, it is likely that OxyR directly regulated *phnW* expression level through binding on an upstream sequence of this gene in certain condition to help protect cell from *t*-BOOH toxicity (**Figs 3 and 4**). Frustratingly, though the mechanism of PhnW in responding to *t*-BOOH or regulated by OxyR is still unclear, this study clearly indicates that this protein has the ability to assist in the protection of *PA* cell from *t*-BOOH toxicity.

Given the frustrating caveat of not knowing the true mechanism by which PhnW influences *t*-BOOH resistance not directly but possibly indirectly, we propose the following model of *phnW* expression under different cellular conditions that help protect bacteria from *t*-BOOH. A cartoon depicting various oxidative conditions in selected strains is shown in **Fig 7A–7D**. First, there is continuous expression of *phnW* in wild-type bacteria (**Fig 7A**).

## Conclusions

In this study, we revealed a previously unrecognized gene under the control of PA OxyR that plays an as yet unknown role in detoxification of the organic hydroperoxide, *t*-BOOH. We expose a dynamic that involves a complex interplay between PhnW, that does not appear to have the direct capacity to degrade *t*-BOOH, but an absence of it in the bacteria shifts the overall expression of other classical AHPs including AhpCF and Ohr. Further studies are required to elucidate the precise detoxification mechanism that PhnW provides to aerobically grown PA.

## Supporting information

S1 Fig Conservation of the OxyR putative binding sequences in several OxyR-regulated genes. The OxyR putative binding region of several OxyR-dependent genes based on (18) was used to compare to OxyR binding region of *phnW*. The MView software from EMBL-EBI was used. The conservation of these sequences in OxyR-dependent genes such as *phnW*, *betX*, *ahpB*, *katB*, *ahpC* and *bdlA* are shown in black. Dark gray represent the nucleotides homologous to the OxyR putative binding region of *phnW* while lighter gray are not.
(TIFF)

S2 Fig KEGG map of the location of the PhnW protein in normal metabolism of *PA*. **A.** Gene orientation of the *phnW* gene on the *PA* PAO1 genome. **B.** KEGG map of a portion of central metabolism of *PA* with the PhnW protein catalyzing the reaction shown as an arrow. The black arrows on the upper left corner of the diagram indicate the potential flow of acetate and acetaldehyde back in the glycolytic pathway.
(TIFF)
