# Supplementary figures and images for: The OxyR-regulated phnW gene encoding 2-aminoethylphosphonate:pyruvate aminotransferase helps protect Pseudomonas aeruginosa from tert-butyl hydroperoxide
Source: PLoS One. 2017 Dec 7;12(12):e0189066. doi: 10.1371/journal.pone.0189066 (PMC5720770; doi:10.1371/journal.pone.0189066)

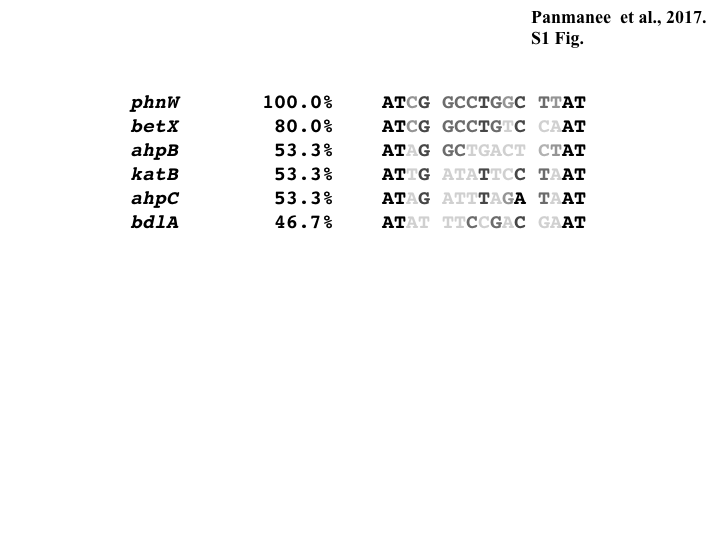

Supplement: S1 Fig — The OxyR putative binding region of several OxyR-dependent genes based on (18) was used to compare to OxyR binding region of phnW. The MView software from EMBL-EBI was used. The conservation of these sequences in OxyR-dependent genes such as phnW, betX, ahpB, katB, ahpC and bdlA are shown in black. Dark gray represent the nucleotides homologous to the OxyR putative binding region of phnW while lighter gray are not. (TIFF) [file pone.0189066.s001.tiff]

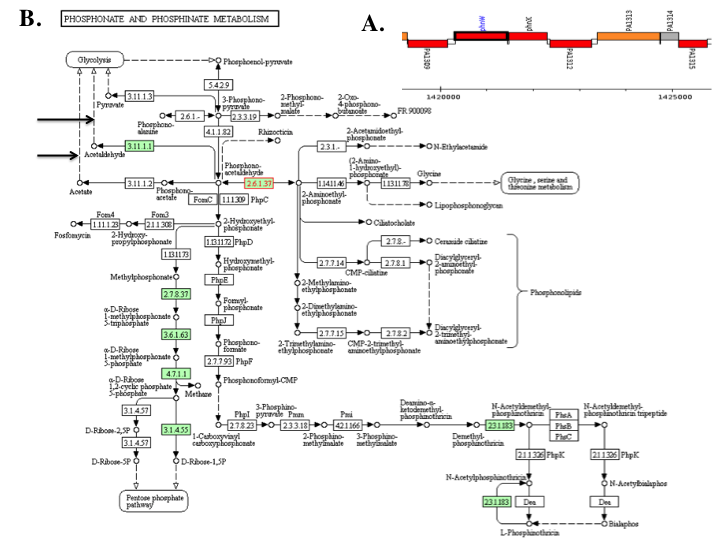

Supplement: S2 Fig — A. Gene orientation of the phnW gene on the PA PAO1 genome. B. KEGG map of a portion of central metabolism of PA with the PhnW protein catalyzing the reaction shown as an arrow. The black arrows on the upper left corner of the diagram indicate the potential flow of acetate and acetaldehyde back in the glycolytic pathway. (TIFF) [file pone.0189066.s002.tiff]
